# Supplementary material for: Glutamate helps unmask the differences in driving forces for phase separation versus clustering of FET family proteins in sub-saturated solutions
Source: Res Sq. 2023 Sep 18:rs.3.rs-3252197. Preprint. [Version 1] doi: 10.21203/rs.3.rs-3252197/v1 (PMC10543311; doi:10.21203/rs.3.rs-3252197/v1)
Supplement: Supplement 1 [file NIHPPrs3252197v1-supplement-1.pdf]

SUPPLEMENTARY INFORMATION FOR:

Glutamate helps unmask the differences in driving forces for phase separation versus clustering of FET family proteins in sub-saturated solutions

Mrityunjoy Kar [1], Laura T. Vogel [2], Gaurav Chauhan [3], Hannes Ausserwöger [4], Timothy J. Welsh [4], Anjana R. Kamath [1], Tuomas P. J. Knowles [4], Anthony A. Hyman [1, *], Claus A. M. Seidel [2, *], and Rohit V. Pappu [3, *]

[1] Max Planck Institute of Cell Biology and Genetics, 01307, Dresden, Germany

[2] Department of Molecular Physical Chemistry, Heinrich Heine University, 40225, Düsseldorf, Germany

[3] Department of Biomedical Engineering and Center for Biomolecular Condensates, Washington University in St. Louis, St. Louis, MO 63130, USA

[4] Centre for Misfolding Diseases, Yusuf Hamied Department of Chemistry, University of Cambridge, CB2 1EW, Cambridge, UK

*e-mail: hyman@mpi-cbg.de, cseidel@hhu.de, pappu@wustl.edu

Section B. Materials

Table S1: List of reagents, sources, and vendor identifiers if any

REAGENTS	SOURCE	IDENTIFIER
**CHEMICALS**		
TRIS	Carl Roth Germany	77-86-1
Potassium Chloride (KCl)	Merck Germany	7447-40-7
Potassium Glutamate (KGlu)	Merck Germany	6382-01-0
Glutamic Acid	Merck Germany	56-86-0
Glycerol	VWR chemicals	56-81-5
cOmplete™	Roche Germany	11697498001
Imidazole	Merck Germany	288-32-4
DTT	Alfa Aesar Germany	578-51-7
Maltose	Merck Germany	6363-53-7
Hydrochloric Acid	Merck Germany	7647-01-0
Bis-ANS	Merck Germany	65664-81-5
Nile Red	Merck Germany	7385-67-3
**Bacterial and Virus Strains**		
Sf9 cells	Expression Systems	Cat#94-001F
**Recombinant proteins**		
FUS-EGFP	Wang et. al. 2018 [1]	TH1204
FUS-SNAP	Wang et. al. 2018 [1]	TH0901
FUS	Wang et. al. 2018 [1]	TH0901
FUS (RBD, 24R-K)	Wang et. al. 2018 [1]	TH1149
FUS (RBD, 24R-G)	Wang et. al. 2018 [1]	TH1006
FUS (RBD, 10D/4E -G)	Kar et. al. 2022 [2]	TH1740
FUS (PLD,10Y-S)	Kar et. al. 2022 [2]	TH1427
FUS (PLD, 18Y-S)	Kar et. al. 2022 [2]	TH1815

FUS (PLD, 27Y-S)	Wang et. al. 2018 [1]	TH0992
FUS (RBD, 6F-G)	Kar et. al. 2022 [2]	TH1917
FUS (RBD, 6Y-S)	Kar et. al. 2022 [2]	TH1918
TAF15-SNAP	Wang et. al. 2018 [1]	TH1203
EWSR1-SNAP	Wang et. al. 2018 [1]	TH1276

Buffers for the experiments:

For buffers, we prepared the following stock solutions;

i) 2 M KCl

ii) 1 M TRIS.HCl pH 7.4 (1 M TRIS was solubilized in DI water and added HCl to adjust the pH to 7.4).

iii) 2 M KGlu

iv) 1 M TRIS.Glu pH 7.4 (1 M TRIS was solubilized in DI water and added Glutamic acid to adjust the pH to 7.4)

Final buffer composition for protein-based experiments:

100 mM KCl buffer: The KCl amount was calculated from the residual KCl from the protein stock solution and added to yield a 100 mM final concentration for each experiment. The buffer also contains 20 mM TRIS. HCl pH 7.4.

KCl-control buffer: The 1x buffer consists of 20 mM TRIS. HCl pH 7.4 and 100 mM KCl. However, in each experiment, the final KCl was not constant as residual KCl from protein stock was added.

KGlu buffer: The 1x buffer consists of 20 mM TRIS. Glu pH 7.4 and 100 mM KGlu. In each experiment, the residual KCl from protein stock was added.

Constructs, protein expression and purification:

The construct/protein sequences used are listed in Section C. Details of materials used for the preparation of samples are as follows:

***Lysis buffer*:** 50 mM TRIS. HCl pH 7.4, 1 M KCl, and 5% Glycerol.

***Protease inhibitor*:** cOmplete™, EDTA-free Protease Inhibitor Cocktail Tablets.

***NTA elution buffer*:** 50 mM TRIS.HCl pH 7.4, 1 M KCl, 5% Glycerol and 300 mM Imidazole.

***MPB elution buffer*:** 50 mM TRIS.HCl pH 7.4, 1 M KCl, 5% Glycerol and 30 mM Maltose.

***Storage buffe*r:** 50 mM TRIS.HCl pH 7.4, 500 mM KCl, 5% Glycerol, and 1 mM DTT.

Table S1: FRET correction parameters

Correction parameter [3]		FRET pair
**donor**	**D**	**FUS-SNAP-AF488**
**acceptor**	**A**	**FUS-SNAP-AF647**
crosstalk	α	0.010
direct excitation		0.020
detection efficiency ratio	$g_{G\|D} / g_{R\|A}$	0.800
donor fluorescence quantum yield	$\Phi_{F,D(0)}$	0.800
acceptor fluorescence quantum yield	$\Phi_{F,A}$	0.330
acceptor-donor intensity ratio	δ	1.000
green background [kHz]	B_g	1.650
red background [kHz]	B_r	0.707
yellow background [kHz]	B_y	0.672

Table S2: FCS fit parameters

Fit parameter		100 mM KCl	100 mM KGlu
χ^2	***chi-squared***	**1.365**	**40.67**
G_0	*correlation offset*	1.000	0.999
N	*number of molecules in focus*	0.877	0.872
$t_{d1,global}$	*first diffusion time (global) [ms]*	0.214	0.214
$z_{0,1}/\omega_{0,1}$	*focus ratio for* $t_{d1,global}$	1.983	2.090
t_{d2}	*second diffusion time [ms]*	1.055	2.260
$z_{0,2}/\omega_{0,2}$	*focus ratio for* t_{d2}	6.125	598.4
R	*amplitude for* $t_{d1,global}$	0.707	0.718
A	*amplitude for bunching term*	0.076	0.089
$t_{A,global}$	*bunching time (global) [ms]*	0.003	0.003

Section D. Amino Acid Sequences of Proteins used in Spectroscopic Studies

1. FUS-EGFP: This sequence includes full-length FUS (unshaded), a linker that is cleavable by a TEV protease (shaded in yellow), and the EGFP (shaded in green).

```
MASNDYTQQATQSYGAYPTQPGQGYSQQSSQPYGQQSYSGYSQSTDTSGYGQSSYSSYGQSQ
NTGYGTQSTPQGYGSTGGYGSSQSSQSSYGQQSSYPGYGQQPAPSSTSGSYGSSSQSSSYGQ
PQSGSYSQQPSYGGQQQSYGQQQSYNPPQGYGQQNQYNSSSGGGGGGGGGGNYGQDQSSMSS
GGGSGGGYGNQDQSGGGGSGGYGQQASDRGGRGGSGGGGGGGGGGGGYNRSSGGYEPRGRGG
GRGGRGGMGGSDRGGFNKFGGPRDQGSRHDSEQDNSDNNTIFVQGLGENVTIESVADYFKQI
GIIKTNKKTGQPMINLYTDRETGKKGEATVSFDDPPSAKAAIDWFDGKEFSGNPIKVSFATR
RADFNRGGGNGRGGRGRGMGRGGYGGGGSGGGRGGFPSGGGGGGGGQQRAGDWKCPNPTCEN
MNFSWRNECNQCKAPKPDGPGGGPGGSHMGGNYGDDRRGGRGGYDRGGYRGRGGDRGGFRGG
RGGGDRGGFGPGKMDSRGEHRQDRRERPYGAPGSSSGRENLYFQGMVSKGEELFTGVVPILV
ELDGDVNGHKFSVSGEGEGDATYGKLTLKFICTTGKLPVPWPTLVTTLTYGVQCFSRYPDHM
KQHDFFKSAMPEGYVQERTIFFKDDGNYKTRAEVKFEGDTLVNRIELKGIDFKEDGNILGHK
LEYNYNSHNVYIMADKQKNGIKVNFKIRHNIEDGSVQLADHYQQNTPIGDGPVLLPDNHYLS
TQSALSKDPNEKRDHMVLLEFVTAAGITLGMDELYK
```

2. FUS-SNAP: This sequence includes full-length FUS (unshaded), a linker that is cleavable by a TEV protease (shaded in yellow), and the SNAP (shaded in gray).

```
MASNDYTQQATQSYGAYPTQPGQGYSQQSSQPYGQQSYSGYSQSTDTSGYGQSSYSSYGQSQ
NTGYGTQSTPQGYGSTGGYGSSQSSQSSYGQQSSYPGYGQQPAPSSTSGSYGSSSQSSSYGQ
PQSGSYSQQPSGGQQQSYGQQQSYNPPQGYGQQNQYNSSSGGGGGGGGGGNYGQDQSSMSSG
GGSGGGYGNQDQSGGGGSGGYGQQASDRGGRGGSGGGGGGGGGGGGYNRSSGGYEPRGRGGG
RGGRGGMGGSDRGGFNKFGGPRDQGSRHDSEQDNSDNNTIFVQGLGENVTIESVADYFKQIG
```

IIKTNKKTGQPMINLYTDRETGKLKGEATVSFDDPPSAKAAIDWFDGKEFSGNPIKVSFATR
RADFNRGGGNGRGGRGRGGPMGRGGYGGGGSGGGGRGGFPSGGGGGGGQQRAGDWKCPNPTC
ENMNFSWRNECNQCKAPKPDGPGGGPGGSHMGGNYGDDRRGGRGGYDRGGYRGRGGDRGGFR
GGRGGGDRGGFGPGKMDSRGEHRQDRRERPYGAPGSSSGRENLYFQGMDKDCEMKRTTLDSP
LGKLELSGCEQGLHRIIFLGKGTSAADAVEVPAPAAVLGGPEPLMQATAWLNAYFHQPEAIE
EFPVPALHHPVFQQESFTRQVLWKLLKVVKFGEVISYSHLAALAGNPAATAAVKTALSGNPV
PILIPCHRVVQGDLDVGGYEGGLAVKEWLLAHEGHRLGKPGLG

3. FUS

MASNDYTQQATQSYGAYPTQPGQGYSQQSSQPYGQQSYSGYSQSTDTSGYGQSSYSSYGQSQ
NTGYGTQSTPQGYGSTGGYGSSQSSQSSYGQQSSYPGYGQQPAPSSTSGSYGSSSQSSSYGQ
PQSGSYSQQPSYGGQQQSYGQQQSYNPPQGYGQQNQYNSSSGGGGGGGGGNYGQDQSSMSS
GGGSGGGYGNQDQSGGGGSGGYGQQASDRGGRGRGGSGGGGGGGGGGYNRSSGGYEPRGRGG
GRGGRGGMGGSDRGGFNKFGGPRDQGSRHDSEQDNSDNNTIFVQGLGENVTIESVADYFKQI
GIIKTNKKTGQPMINLYTDRETGKLKGEATVSFDDPPSAKAAIDWFDGKEFSGNPIKVSFAT
RRADFNRGGGNGRGGRGRGGPMGRGGYGGGGSGGGGRGGFPSGGGGGGGQQRAGDWKCPNPT
CENMNFSWRNECNQCKAPKPDGPGGGPGGSHMGGNYGDDRRGGRGGYDRGGYRGRGGDRGGF
RGGRGGGDRGGFGPGKMDSRGEHRQDRRERPY

4. FUS(R-K): Full-length FUS with 24 Arg substituted to Lys in the RBD

MASNDYTQQATQSYGAYPTQPGQGYSQQSSQPYGQQSYSGYSQSTDTSGYGQSSYSSY
GQSQNTGYGTQSTPQGYGSTGGYGSSQSSQSSYGQQSSYPGYGQQPAPSSTSGSYGSS
SQSSSYGQPQSGSYSQQPSYGGQQQSYGQQQSYNPPQGYGQQNQYNSSSGGGGGGGGG
GNYGQDQSSMSSGGGSGGGYGNQDQSGGGGSGGYGQQASDKGGKGKGGSGGGGGGGGG
GYNRSSGGYEPKGKGGGKGGKGGMGGSDKGGFNKFGGPRDQGSRHDSEQDNSDNNTIF
VQGLGENVTIESVADYFKQIGIIKTNKKTGQPMINLYTDRETGKLKGEATVSFDDPPS
AKAAIDWFDGKEFSGNPIKVSFATRRADFNKGGGNGKGGKGKGGPMGKGGYGGGGSGG
GGKGGFPSGGGGGGGQQRAGDWKCPNPTCENMNFSWRNECNQCKAPKPDGPGGGPGGS
HMGGNYGDDRKGGKGGYDKGGYKGKGGDKGGFRGGRGGGDRGGFGPGKMDSRGEHRQD
RRERPY

5. FUS(R-G): Full-length FUS with 24 Arg substituted to Gly in the RBD

MASNDYTQQATQSYGAYPTQPGQGYSQQSSQPYGQQSYSGYSQSTDTSGYGQSSYSSYGQSQ
NTGYGTQSTPQGYGSTGGYGSSQSSQSSYGQQSSYPGYGQQPAPSSTSGSYGSSSQSSSYGQ
PQSGSYSQQPSYGGQQQSYGQQQSYNPPQGYGQQNQYNSSSGGGGGGGGGNYGQDQSSMSS
GGGSGGGYGNQDQSGGGGSGGYGQQASDGGGGGGGGSGGGGGGGGGGYNRSSGGYEPGGGGG
GGGGGGGMGGSDGGGFNKFGGPRDQGSRHDSEQDNSDNNTIFVQGLGENVTIESVADYFKQI
GIIKTNKKTGQPMINLYTDRETGKLKGEATVSFDDPPSAKAAIDWFDGKEFSGNPIKVSFAT
RRADFNGGGGNGGGGGGGGGPMGGGGYGGGGSGGGGGGGFPSGGGGGGGQQRAGDWKCPNPT
CENMNFSWRNECNQCKAPKPDGPGGGPGGSHMGGNYGDDRGGGGGGYDGGGYGGGGGDGGGF
GGGGGGGDGGGFGPGKMDSGGEHRQDRRERPY

6. FUS-(10D/4E-G): Full-length FUS with 10 Asp and 4 Glu residues substituted to Gly in the RBD

MASNDYTQQATQSYGAYPTQPGQGYSQQSSQPYGQQSYSGYSQSTDTSGYGQSSYSSYGQSQ
NTGYGTQSTPQGYGSTGGYGSSQSSQSSYGQQSSYPGYGQQPAPSSTSGSYGSSSQSSSYGQ
PQSGSYSQQPSYGGQQQSYGQQQSYNPPQGYGQQNQYNSSSGGGGGGGGGNYGQDQSSMSS

GGGSGGGYGNQDQSGGGGSGGYGQQASDRGGRGRGGSGGGGGGGGGYNRSSGGYEPRGRGG
GRGGRGGMGGSDRGGFNKFGGPRDQGSRHDSEQDNSDNNTIFVQGLGENVTIESVADYFKQI
GIIKTNKKTGQPMINLYTDRETGKLKGEATVSFDDPPSAKAAIDWFDGKEFSGNPIKVSFAT
RRAGFNRGGGNGRGGRGRGGPMGRGGYGGGGSGGGGRGGFPSGGGGGGGQQRAGGWKCPNPT
CGNMNFSWRNGCNQCKAPKPGGPGGGPGGSHMGGNYGGGRRGGRGGYGRGGYRGRGGGRGGF
RGGRGGGGRGGFGPGKMGSRGGHRQGRRGRPY

7. FUS(10Y-S): Full-length FUS with 10 Tyr residues substituted to Ser in the PLD

MASNDYTQQATQSYGASPTQPGQGYSQQSSQPSGQQSYSGSSQSTDTSGSGQSSYSSSGQSQ
NTGYGTQSTPQGSGSTGGYGSSQSSQSSYGQQSSSPGYGQQPAPSSTSGSYGSSSQSSSYGQ
PQSGSSSQQPSYGGQQQSSGQQQSSNPPQGYGQQNQYNSSSGGGGGGGGGNYGQDQSSMSS
GGGSGGGYGNQDQSGGGGSGGYGQQASDRGGRGRGGSGGGGGGGGGYNRSSGGYEPRGRGG
GRGGRGGMGGSDRGGFNKFGGPRDQGSRHDSEQDNSDNNTIFVQGLGENVTIESVADYFKQI
GIIKTNKKTGQPMINLYTDRETGKLKGEATVSFDDPPSAKAAIDWFDGKEFSGNPIKVSFAT
RRADFNRGGGNGRGGRGRGGPMGRGGYGGGGSGGGGRGGFPSGGGGGGGQQRAGDWKCPNPT
CENMNFSWRNECNQCKAPKPDGPGGGPGGSHMGGNYGDDRRGGRGGYDRGGYRGRGGDRGGF
RGGRGGGDRGGFGPGKMDSRGEHRQDRRERPY

8. FUS(18Y-S): Full-length FUS with 18 Tyr residues substituted to Ser in the PLD

MASNDSTQQATQSYGASPTQPGQGSSQQSSQPSGQQSYSGSSQSTDTSGSGQSSYSSSGQSQ
NTGSGTQSTPQGSGSTGGSGSSQSSQSSYGQQSSSPGYGQQPAPSSTSGSSGSSSQSSSSGQ
PQSGSSSQQPSYGGQQQSSGQQQSSNPPQGYGQQNQSNSSSGGGGGGGGGNSGQDQSSMSS
GGGSGGGYGNQDQSGGGGSGGYGQQASDRGGRGRGGSGGGGGGGGGYNRSSGGYEPRGRGG
GRGGRGGMGGSDRGGFNKFGGPRDQGSRHDSEQDNSDNNTIFVQGLGENVTIESVADYFKQI
GIIKTNKKTGQPMINLYTDRETGKLKGEATVSFDDPPSAKAAIDWFDGKEFSGNPIKVSFAT
RRADFNRGGGNGRGGRGRGGPMGRGGYGGGGSGGGGRGGFPSGGGGGGGQQRAGDWKCPNPT
CENMNFSWRNECNQCKAPKPDGPGGGPGGSHMGGNYGDDRRGGRGGYDRGGYRGRGGDRGGF
RGGRGGGDRGGFGPGKMDSRGEHRQDRRERPY

9. FUS(27Y-S): Full-length FUS with 27 Tyr residues substituted to Ser in the PLD

MASNDSTQQATQSSGASPTQPGQGSSQQSSQPSGQQSSSGSSQSTDTSGSGQSSSSSSGQSQ
NTGSGTQSTPQGSGSTGGSGSSQSSQSSSGQQSSSPGSGQQPAPSSTSGSSGSSSQSSSSGQ
PQSGSSSQQPSSGGQQQSSGQQQSSNPPQGSGQQNQSNSSSGGGGGGGGGNSGQDQSSMSS
GGGSGGGSGNQDQSGGGGSGGSGQQASDRGGRGRGGSGGGGGGGGGYNRSSGGYEPRGRGG
GRGGRGGMGGSDRGGFNKFGGPRDQGSRHDSEQDNSDNNTIFVQGLGENVTIESVADYFKQI
GIIKTNKKTGQPMINLYTDRETGKLKGEATVSFDDPPSAKAAIDWFDGKEFSGNPIKVSFAT
RRADFNRGGGNGRGGRGRGGPMGRGGYGGGGSGGGGRGGFPSGGGGGGGQQRAGDWKCPNPT
CENMNFSWRNECNQCKAPKPDGPGGGPGGSHMGGNYGDDRRGGRGGYDRGGYRGRGGDRGGF
RGGRGGGDRGGFGPGKMDSRGEHRQDRRERPY

10. FUS(6F-G): Full-length FUS with 6 Phe residues substituted to Gly in the RBD

AMASNDYTQQATQSYGAYPTQPGQGYSQQSSQPYGQQSYSGYSQSTDTSGYGQSSYSSYGQS
QNTGYGTQSTPQGYGSTGGYGSSQSSQSSYGQQSSYPGYGQQPAPSSTSGSYGSSSQSSSYG
QPQSGSYSQQPSYGGQQQSYGQQQSYNPPQGYGQQNQYNSSSGGGGGGGGGNYGQDQSSMS
SGGGSGGGYGNQDQSGGGGSGGYGQQASDRGGRGRGGSGGGGGGGGGYNRSSGGYEPRGRG
GGRGGRGGMGGSDRGGGNKGGGPRDQGSRHDSEQDNSDNNTIFVQGLGENVTIESVADYFKQ
IGIIKTNKKTGQPMINLYTDRETGKLKGEATVSFDDPPSAKAAIDWFDGKEFSGNPIKVSFA
TRRADGNRGGGNGRGGRGRGGPMGRGGYGGGGSGGGGRGGGPSGGGGGGGQQRAGDWKCPNP

TCENMNFSWRNECNQCKAPKPDGPGGGPGGSHMGGNYGDDRRGGRGGYDRGGYRGRGGDRGG
GRGGRGGGDRGGGGPGKMDSRGEHRQDRRERPY

11. FUS(6Y-S): Full-length FUS with 6 Tyr residues substituted to Ser in the RBD

AMASNDYTQQATQSYGAYPTQPGQGYSQQSSQPYGQQSYSGYSQSTDTSGYGQSSYSSYGQS
QNTGYGTQSTPQGYGSTGGYGSSQSSQSSYGQQSSYPGYGQQPAPSSTSGSYGSSSQSSSYG
QPQSGSYSQQPSYGGQQQSYGQQQSYNPPQGYGQQNQYNSSSGGGGGGGGGGNYGQDQSSMS
SGGGSGGGYGNQDQSGGGGSGGYGQQASDRGGRGRGGSGGGGGGGGGGSNRSSGGSEPRGRG
GGRGGRGGMGGSDRGGFNKFGGPRDQGSRHDSEQDNSDNNTIFVQGLGENVTIESVADYFKQ
IGIIKTNKKTGQPMINLYTDRETGKLKGEATVSFDDPPSAKAAIDWFDGKEFSGNPIKVSFA
TRRADFNRGGGNGRGGRGRGGPMGRGGSGGGGSGGGGRGGFPSGGGGGGGQQRAGDWKCPNP
TCENMNFSWRNECNQCKAPKPDGPGGGPGGSHMGGNSGDDRRGGRGGSDRGGSRGRGGDRGG
FRGGRGGGDRGGFGPGKMDSRGEHRQDRRERPY

12. Taf15-SNAP: This sequence includes full-length Taf15 (unshaded), a linker that is cleavable by a TEV protease (shaded in yellow), and the SNAP tag (shaded in gray).

MSDSGSYGQSGGEQQSYSTYGNPGSQGYGQASQSYSGYGQTTDSSYGQNYSGYSSYGQSYSQ
SYGGYENQKQSSYSQQPYNNQGQQQNMESSGSQGGRAPSYDQPDYGQQDSYDQQSGYDQHQG
SYDEQSNYDQQHDSYSQNQQSYHSQRENYSHHTQDDRRDVSRYGEDNRGYGGSQGGGRGRGG
YDKDGRGPMTGSSGGDRGGFKNFGGHRDYGPRTDADSESDNSDNNTIFVQGLGEGVSTDQVG
EFFKQIGIIKTNKKTGKPMINLYTDKDTGKPKGEATVSFDDPPSAKAAIDWFDGKEFHGNII
KVSFATRRPEFMRGGGSGGGRRGRGGYRGRGGFQGRGGDPKSGDWVCPNPSCGNMNFARRNS
CNQCNEPRPEDSRPSGGDFRGRGYGGERGYRGRGGRGGDRGGYGGDRSGGGYGGDRSSGGGY
SGDRSGGGYGGDRSGGGYGGDRGGGYGGDRGGGYGGDRGGGYGGDRGGYGGDRGGGYGGDRG
GYGGDRGGYGGDRGGYGGDRGGYGGDRSRGGYGGDRGGGSGYGGDRSGGYGGDRSGGGYGGD
RGGGYGGDRGGYGGKMGGRNDYRNDQRNRPYGAPGSSSGRENLYFQGMDKDCEMKRTTLDSP
LGKLELSGCEQGLHRIIFLGKGTSAADAVEVPAPAAVLGGPEPLMQATAWLNAYFHQPEAIE
EFPVPALHHPVFQQESFTRQVLWKLLKVVKFGEVISYSHLAALAGNPAATAAVKTALSGNPV
PILIPCHRVVQGDLDVGGYEGGLAVKEWLLAHEGHRLGKPGLG

13. Ewsr1-SNAP: This sequence includes full-length Ewsr1 (unshaded), a linker that is cleavable by a TEV protease (shaded in yellow), and the SNAP tag (shaded in gray).

MASTDYSTYSQAAAQQGYSAYTAQPTQGYAQTTQAYGQQSYGTYGQPTDVSYTQAQTTATYG
QTAYATSYGQPPTGYTTPTAPQAYSQPVQGYGTGAYDTTTATVTTTQASYAAQSAYGTQPAY
PAYGQQPAATAPTRPQDGNKPTETSQPQSSTGGYNQPSLGYGQSNYSYPQVPGSYPMQPVTA
PPSYPPTSYSSTQPTSYDQSSYSQQNTYGQPSSYGQQSSYGQQSSYGQQPPTSYPPQTGSYS
QAPSQYSQQSSSYGQQSSFRQDHPSSMGVYGQESGGFSGPGENRSMSGPDNRGRGRGGFDRG
GMSRGGRGGGRGGMGSAGERGGFNKPGGPMDEGPDLDLGPPVDPDEDSDNSAIYVQGLNDSV
TLDDLADFFKQCGVVKMNKRTGQPMIHIYLDKETGKPKGDATVSYEDPPTAKAAVEWFDGKD
FQGSKLKVSLARKKPPMNSMRGGLPPREGRGMPPPLRGGPGGPGGPGGPMGRMGGRGGDRGG
FPPRGPRGSRGNPSGGGNVQHRAGDWQCPNPGCGNQNFAWRTECNQCKAPKPEGFLPPPFPP
PGGDRGRGGPGGMRGGRGGLMDRGGPGGMFRGGRGGDRGGFRGGRGMDRGGFGGGRRGGPGG
PPGPLMEQMGGRRGGRGGPGKMDKGEHRQERRDRPYGAPGSSSGRENLYFQGMDKDCEMKRT
TLDSPLGKLELSGCEQGLHRIIFLGKGTSAADAVEVPAPAAVLGGPEPLMQATAWLNAYFHQ
PEAIEEFPVPALHHPVFQQESFTRQVLWKLLKVVKFGEVISYSHLAALAGNPAATAAVKTAL
SGNPVPILIPCHRVVQGDLDVGGYEGGLAVKEWLLAHEGHRLGKPGLG

References

1. Wang J, Choi J-M, Holehouse AS, Lee HO, Zhang X, Jahnel M*, et al.* A Molecular Grammar Governing the Driving Forces for Phase Separation of Prion-like RNA Binding Proteins. *Cell* 2018, **174**(3)**:** 688-699. e616.

2. Kar M, Dar F, Welsh TJ, Vogel LT, Kühnemuth R, Majumdar A*, et al.* Phase-separating RNA-binding proteins form heterogeneous distributions of clusters in subsaturated solutions. *Proceedings of the National Academy of Sciences* 2022, **119**(28)**:** e2202222119.

3. Kudryavtsev V, Sikor M, Kalinin S, Mokranjac D, Seidel CAM, Lamb DC. Combining MFD and PIE for Accurate Single-Pair Förster Resonance Energy Transfer Measurements. *ChemPhysChem* 2012, **13**(4)**:** 1060-1078.
